# Endothelial bioreactor system ameliorates multiple organ dysfunction in septic rats

**DOI:** 10.1186/s40635-016-0097-y

**Published:** 2016-07-22

**Authors:** Shuai Ma, Yuli Lin, Bo Deng, Yin Zheng, Chuanming Hao, Rui He, Feng Ding

**Affiliations:** Division of Nephrology & Unit of Critical Nephrology, Shanghai Ninth People’s Hospital, School of Medicine, Shanghai Jiaotong University, 639 Zhizaoju Road, Shanghai, 200011 China; Department of Immunology, Shanghai Medical College, Fudan University, Shanghai, 200032 China; Division of Nephrology, Huashan Hospital, Fudan University, Shanghai, 200040 China

**Keywords:** Sepsis, Multiple organ dysfunction, Endothelial cells, Bioreactor, Extracorporeal circulation, Neutrophils, Inflammation

## Abstract

**Background:**

The endothelium is a potentially valuable target for sepsis therapy. We have previously studied an extracorporeal endothelial cell therapy system, called the endothelial bioreactor (EBR), which prolonged the survival time of endotoxemia sepsis in swine. To further study of the therapeutic effects and possible mechanisms, we established a miniature EBR system for septic rats induced by cecal ligation and puncture (CLP).

**Methods:**

In the miniature EBR system, the extracorporeal circulation first passed through a mini-hemofilter, and the ultrafiltrate (UF) was separated, then the UF passed through an EBR (a 1-mL cartridge containing approximately 2 × 10^6^ endothelial cells grown on microcarriers) and interact with endothelial cells. Eighteen hours after CLP, the rats were treated for 4 h with this extracorporeal system containing either endothelial cells (EBR group) or no cells (sham EBR group). Physiologic and biochemical parameters, cytokines, endothelial functions, and 7-day survival time were monitored. In vitro, the pulmonary endothelial cells of the septic rats were treated with the EBR system and the resulting changes in their functions were monitored.

**Results:**

The EBR system ameliorated CLP-induced sepsis compared with the sham EBR system. After CLP, the 7-day survival rate of sham-treated rats was only 25.0 %, while in the EBR-treated group, it increased to 57.1 % (*p* = 0.04). The EBR system protected the liver and renal function and ameliorated the kidney and lung injury. Meanwhile, this therapy reduced pulmonary vascular leakage and alleviated the infiltration of inflammatory cells in the lungs, especially neutrophils. Furthermore, after the EBR treatment both in vivo and in vitro, the expression of intercellular adhesion molecule-1 and the secretion of CXCL1 and CXCL2 of pulmonary endothelium decreased, which helped to alleviate the adhesion and chemotaxis of neutrophils. In addition, the EBR system decreased CD11b expression and intracellular free calcium level of peripheral blood neutrophils, modulated the activation of these neutrophils.

**Conclusions:**

The EBR system significantly ameliorated CLP-induced sepsis and improved survival and organ functions. Compared with the sham EBR system, this extracorporeal endothelial therapy may be involved in modulating the function of pulmonary endothelial cells, reducing the adhesion and chemotaxis of neutrophil, and modulating the activation of peripheral blood neutrophils.

**Electronic supplementary material:**

The online version of this article (doi:10.1186/s40635-016-0097-y) contains supplementary material, which is available to authorized users.

## Background

Sepsis is defined as life-threatening organ dysfunction caused by a dysregulated host response to infection [1] and is the most common cause of death in the intensive care unit [2]. Despite the progress in the development of antibiotics and in critical care therapy, sepsis is still associated with a high mortality rate [2].

Cell-based therapy has developed into a new therapeutic platform for the treatment of a vast array of clinical disorders, which may prove to be a more successful strategy by providing a dynamic, interactive, and individualized therapeutic approach [3]. In animal studies, stem/progenitor technologies, including exogenous infusion and endogenous recruitment, have shown significant promise as treatment strategies in critical care medicine [4–7]. In terms of safety, extracorporeal cell therapies may be a better choice, which not only replace the function of injury cells or modulate the pathophysiological processes but also provide an immunoprotective barrier. In swine models of sepsis, extracorporeal cell therapy with granulocytes [8] or renal proximal tubule cells [9] improved survival duration. Furthermore, in a phase II multicenter clinical trial, involving critically ill patients with acute renal failure, this extracorporeal renal-tubule cell therapy was proved to be efficacious and safe [10]. Nevertheless, the difficulties in large animal experiments keep the mechanism study elucidation insufficient.

General dysfunction of the endothelium is a key event in the pathogenesis of sepsis [11]. Once the endothelium becomes activated during the development of sepsis, it transforms into a procoagulant, antifibrinolytic, and proadhesive state [12]. The endothelial activation in sepsis is associated with changes in hemostatic balance, leukocyte trafficking, vascular permeability, inflammatory processes, and microcirculatory flow [13]. Thus, the endothelium plays a key role in mediating the sepsis phenotype [14] and is a potentially valuable target of sepsis therapy.

We previously studied an endothelial cell therapy system [15], called the endothelial bioreactor (EBR), involving a nonwoven fabric polytetrafluoroethylene (PTFE) hollow fiber cartridge containing endothelial cells in an extracorporeal circuit. Timely use of EBR therapy may improve cardiovascular performance and prolong the survival time of endotoxemia sepsis in swine [15]. Nevertheless, the difficulties in large animal experiments keep its elucidation insufficient. Therefore, we established a miniature EBR system for septic rats to study the therapeutic effects and possible mechanisms of this endothelial cell-based therapy.

## Methods

### Cecal ligation and puncture

Adult male Sprague-Dawley rats (450–550 g) were used for the studies. The experimental procedures were in accordance with the Animal Care and Use Committee of Shanghai Jiaotong University, Shanghai, China. All surgical procedures were carried out under general anesthesia induced by 4 % chloral hydrate (0.9 mL/100 g intraperitoneally). The cecum was identified and ligated with a 3-0 silk tie at 25 % length of the cecum. A double puncture of the cecal wall was performed with a 20-gauge needle, and the cecum was gently squeezed to ensure that a small amount of feces was extruded onto the surface of the bowel. Lactated Ringer’s solution (2 mL/100 g) was given subcutaneously as fluid resuscitation [16, 17]. As sham cecal ligation and puncture (CLP) rats, the cecum was minimally handled without ligation and puncture.

### Culturing of endothelial cells on microcarriers

Endothelial cells (human umbilical vein endothelial cells (HUVECs)) were a gift from Professor Lijun Jia (Department of Immunology, Shanghai Medical College, Fudan University, Shanghai, China).

For endothelial cell culture, protocols were adapted based on the recommendations for microcarrier culture using Cytodex-3 (GE Healthcare, Piscataway, USA). Cytodex-3 is a microporous microcarrier, made up of a dextran matrix with a collagen layer at the surface, for expansion of cells. For stirred microcarrier cultures, 100-mL spinner flasks (Bellco Glass, Vineland, USA) were used, with a final medium volume of 80 mL and a stirring speed of 40 rpm. Required quantities of Cytodex-3 were weighted (final concentrations of 1.0 mg/mL), hydrated, and sterilized by autoclaving as recommended by the manufacturers. Microcarriers were equilibrated in the culture medium for at least 30 min prior to the addition of the cells in order to maximize cell attachment. The cells were cultured in DMEM media containing 10 % heat-inactivated fetal bovine serum (GIBCO, Gaithersburg, USA), 100 U/mL penicillin, 100 μg/mL streptomycin, and 2 mM glutamine. Incubation was conducted at 37 °C in a 5 % CO_2_, humidified atmosphere. The expansion of endothelial cells was performed as previously described [18].

### The experimental protocol of the EBR system

Eighteen hours after CLP, the animals were re-anesthetized with chloral hydrate. The carotid artery and the femoral vein were isolated by dissection and cannulated with 0.97-mm polyethylene-50 tubing (Becton Dickinson, San Diego, USA) for implementation of extracorporeal circulation.

The EBR system consisted of a mini-hemofilter (polyether sulphone high-flux membrane, membrane surface area 0.02 m^2^, cutoff point 40 kD; PEONY, Shanghai, China), an EBR (a 1-mL cartridge containing approximately 2 × 10^6^ endothelial cells grown on microcarriers), tubing lines, and mini-pumps (VWR, West Chester, USA).

**Fig. 1:**
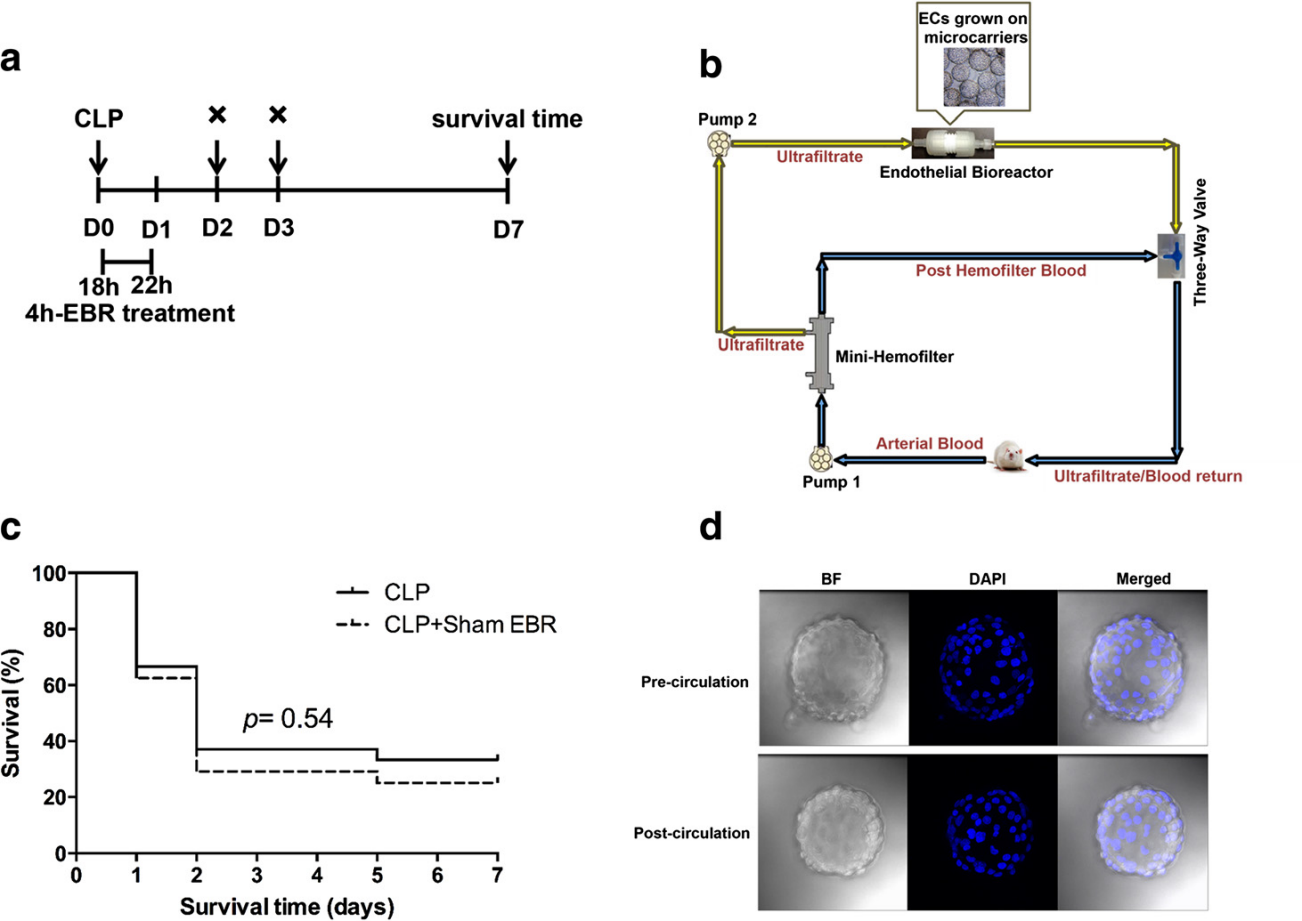
Construction of the endothelial bioreactor (EBR) system for septic rats. **a** The protocol for the EBR treatment. **b** A schematic drawing of the EBR system. **c** Survival was plotted during a 7-day period (*n* = 22–25 per group). **d** Endothelial cells grown on microcarriers were collected before and after the extracorporeal circulation and were stained with DAPI (*blue*) at ×400 ×Z 1.5 magnification. *CLP* cecal ligation and puncture, *EBR* endothelial bioreactor, *ECs* endothelial cells, *DAPI* 4′,6-diamidino-2-phenylindole, *BF* bright field

Eighteen hours after CLP, these animals were randomly assigned to receive either EBR or sham treatment for 4 h. The extracorporeal circulation was driven by a mini-pump from the carotid artery to the femoral vein at a blood flow rate of 0.8–1.0 mL/min [19]. In the EBR group, the extracorporeal circulation passed through a mini-hemofilter with the ultrafiltration rate at 0.25 mL/min, then the ultrafiltrate (UF) passed through an EBR (Fig. 1a, b). In the sham EBR group, the extracorporeal circulation was set up with the same hemofilter and bioreactor cartridge but without any cells. In the control group, all rats were without the CLP procedure or the extracorporeal circulation. A volume of 62.5 U/mL of heparin was used to prevent coagulation in this circuit. After 4 h, the treatment was stopped and the rats were observed for recovery, returned to the animal facility, and given access to food and water. Survival time was assessed up to 7 days. Another group that underwent the shame CLP procedure without extracorporeal circulation was used as the control. Before and after the extracorporeal circulation, microcarriers were collected and stained with 4′,6-diamidino-2-phenylindole (DAPI; Becton Dickinson, San Diego, USA) and observed under a confocal laser scanning microscope (Leica Microsystems, Heidelberg, Germany) at a magnification of ×400 ×Z 1.5.

### Analysis of bronchoalveolar lavage fluid

Bronchoalveolar lavage fluid (BALF) was obtained by lavaging the left lung once with 2 mL PBS and three times with 4 mL PBS. Total cells were counted under optical microscopes. Total protein in the first BALF was measured by bicinchoninic acid kit. TNF-α, IL-1β, and IL-6 levels in the first BALF were measured by an enzyme-linked immunosorbent assay (ELISA) from eBioscience (San Diego, USA).

### Flow cytometric analysis

Fluorochrome-labeled antibodies CD4, CD8, CD25, Foxp3, CD45, CD11b, and Granulocyte Marker (eBioscience, San Diego, USA) were used for surface and intracellular staining, according to the manufacturer’s instructions. After staining, cells were analyzed by flow cytometer (Becton Dickinson, San Diego, USA).

### Quantitative real-time RT-PCR analysis

Total RNA was extracted using Micro Scale RNAqueous Isolation kit, and then cDNA was synthesized with the High-Capacity cDNA Reverse Transcription kit (both from Applied Biosystems, Foster City, USA). Quantitative real-time polymerase chain reaction was performed with the SYBR green Gene Expression Assay (Applied Biosystems, Foster City, USA). The relative expressions of target genes were calculated using the 2^Δ^C(t) method. The sequences of primers for polymerase chain reaction are as follows: intercellular adhesion molecule (ICAM)-1, 5′-CTCATCCTGCGCTGTCTGGT-3′ (forward), 5′-CCGGAGCTGCCTGACCTCGG-3′ (reverse); CXCL1, 5′-GCGTTTCATCGATGGTCGTT-3′ (forward), 5′-CTTCTTCCCGCTCAACACCT-3′ (reverse); and CXCL2, 5′-AACCATCAGGGTACAGGGGT-3′ (forward), 5′-GGGCTTCAGGGTTGAGACAA-3′ (reverse).

### Western blot analysis for ICAM-1

Pulmonary endothelial cells or lung tissue were lysed with RIPA buffer. Proteins were separated by electrophoresis in a denaturing polyacrylamide gel and transferred to a PVDF membrane. After blocking with 5 % milk-Tris-buffered saline and Tween 20 (TBST) and washing in TBST, membranes were then incubated in the appropriate primary antibodies (anti-ICAM-1; both from R&D, Emeryville, USA) and anti-GAPDH (Cell Signal Technology, Beverly, USA) at 4 °C overnight. After washing, membranes were incubated with the appropriate HRP-conjugated secondary antibodies and analyzed by ECL development.

### Isolation of rat pulmonary endothelial cells

The lungs were harvested at 18 h after CLP, finely minced, and digested in 10 ml collagenase type II (2 mg/ml, Sigma-Aldrich, St. Louis, USA). The isolated cells were incubated with Anti-Rat CD31 Biotin (eBioscience, San Diego, USA), and endothelial cells bound with antibody were magnetically separated with Anti-Biotin Microbeads (Miltenyi Biotec, Bergisch Gladbach, Germany) as described previously [20, 21]. Pulmonary endothelial cells of healthy rats were also isolated as control.

### Simulation of EBR treatment in vitro

Eighteen hours after CLP, the rats were cannulated, and the extracorporeal circulation passed through a mini-hemofilter with the ultrafiltration rate at 0.25 mL/min. The UF of the rats were harvested (sham-UF). The UF of healthy rats were also harvested as a control (control-UF). HUVECs were stimulated with the UF for 0, 0.5, 1, 2, or 4 h. Then, the supernatant (EBR-UF) was collected and used to stimulate the isolated pulmonary endothelial cells. After 24 h of stimulation with EBR-UF, the cells were harvested for Western blot and the supernatant was used to analyze the concentrations of CXCL1 and CXCL2 using ELISA kits (R&D, Emeryville, USA).

### Isolation of rat peripheral blood neutrophils and detection of intracellular free calcium level

Rat neutrophils were isolated from the peripheral blood at 18 h after CLP by Percoll density gradient as previously described [22]. The purity of isolated neutrophils was checked by fluorochrome-labeled antibodies CD45 and Granulocyte Marker (eBioscience, San Diego, USA) using flow cytometer (Becton Dickinson, San Diego, USA). Neutrophils of healthy rats were also isolated as a control.

The neutrophils were incubated with Fluo-3 AM (Invitrogen) for 30 min in darkness at room temperature, and the resulting fluorescence as the indicator of Ca^2+^ was monitored using a flow cytometer (Becton Dickinson, San Diego, USA) at excitation wavelength 488 nm and the emission wavelength of 525 nm [23, 24].

### Histopathologic analysis of the lung and kidney

Lung sections were stained with hematoxylin and eosin. All lung fields at ×200 magnification were examined for each sample. Assessment of histological lung injury was performed as follows: 1 = normal; 2 = focal (<50 % lung section) interstitial congestion and inflammatory cell infiltration; 3 = diffuse (>50 % lung section) interstitial congestion and inflammatory cell infiltration; 4 = focal (<50 % lung section) consolidation and inflammatory cell infiltration; 5 = diffuse (>50 % lung section) consolidation and inflammatory cell infiltration [25].

Kidney sections were stained with periodic acid and Schiff’s reagent. All kidney fields at ×400 magnification were examined for each sample. Damaged renal tubules were identified by diffuse tubular dilatation, intraluminal casts and/or tubular cell blebbing, vacuolization, and detachment, in cortex and outer medulla, as follows: 0 = none; 1 = <10 %; 2 = 11–25 %; 3 = 26–45 %; 4 = 46–75 %; 5 = >76 % [26, 27].

### Measurements of the liver and renal functions

Serum alanine aminotransferase (ALT), aspartate aminotransferase (AST), creatinine (Cr), and blood urea nitrogen (BUN) were measured with an automated chemical analyzer (Vitros-950, Johnson & Johnson, New Brunswick, USA).

### Statistical analysis

All numerical data are expressed as mean ± SEM. The Student’s *t* tests were used for comparisons between two groups. Multiple-group comparisons were performed by one-way ANOVA followed by a post hoc Tukey’s test to compare each group. The survival analysis was performed by the Kaplan-Meier method and log-rank test. All statistical analyses were performed with GraphPad Prism software, and a two-sided *p* < 0.05 was considered significant.

## Results

### Construction of the EBR system for CLP-induced sepsis in rats

In order to estimate the potential harmful effect of this extracorporeal circulation on survival time, the rats were divided into two groups: the CLP group (CLP, without extracorporeal circulation) and the sham EBR group (CLP, EBR system without any cells); then, survival was analyzed until 7 days after CLP. We found that survival duration was not statistically different between the sham EBR and control animals (25.0 vs. 33.3 %, *p* = 0.54; Fig. 1c), which meant this circulation system was safe for the septic rats. The EBR was a 1-mL cartridge, whereas we need approximately 2 × 10^6^ endothelial cells in it. Thus, we cultured endothelial cells on microcarriers to increase the surface-area-to-volume ratio. During the culturing of endothelial cells on microcarriers, we analyzed the concentrations of glucose, lactate, and nitric oxide (NO) in different time points for analyzing the metabolic activity. We found that endothelial cells could grow adhering to the surface of microcarriers after seeding; lactate and NO levels in the culture media rose continuously, and the glucose concentration decreased with time, indicating metabolic activity residing in the microcarrier culture (data not shown). For testing whether the EBR system treatment will affect the adhesion of endothelial cells on the microcarrier, we collected microcarriers and stained them with DAPI before and after the extracorporeal circulation. We observed that there were a large number of endothelial cells growing on the microcarriers before the circulation. Similar observations after the extracorporeal circuit revealed no obvious decrease in the density of the cells (Fig. 1d). These data showed that we successfully constructed an EBR system for rats with CLP-induced sepsis.

### The EBR system ameliorated CLP-induced sepsis

**Fig. 2:**
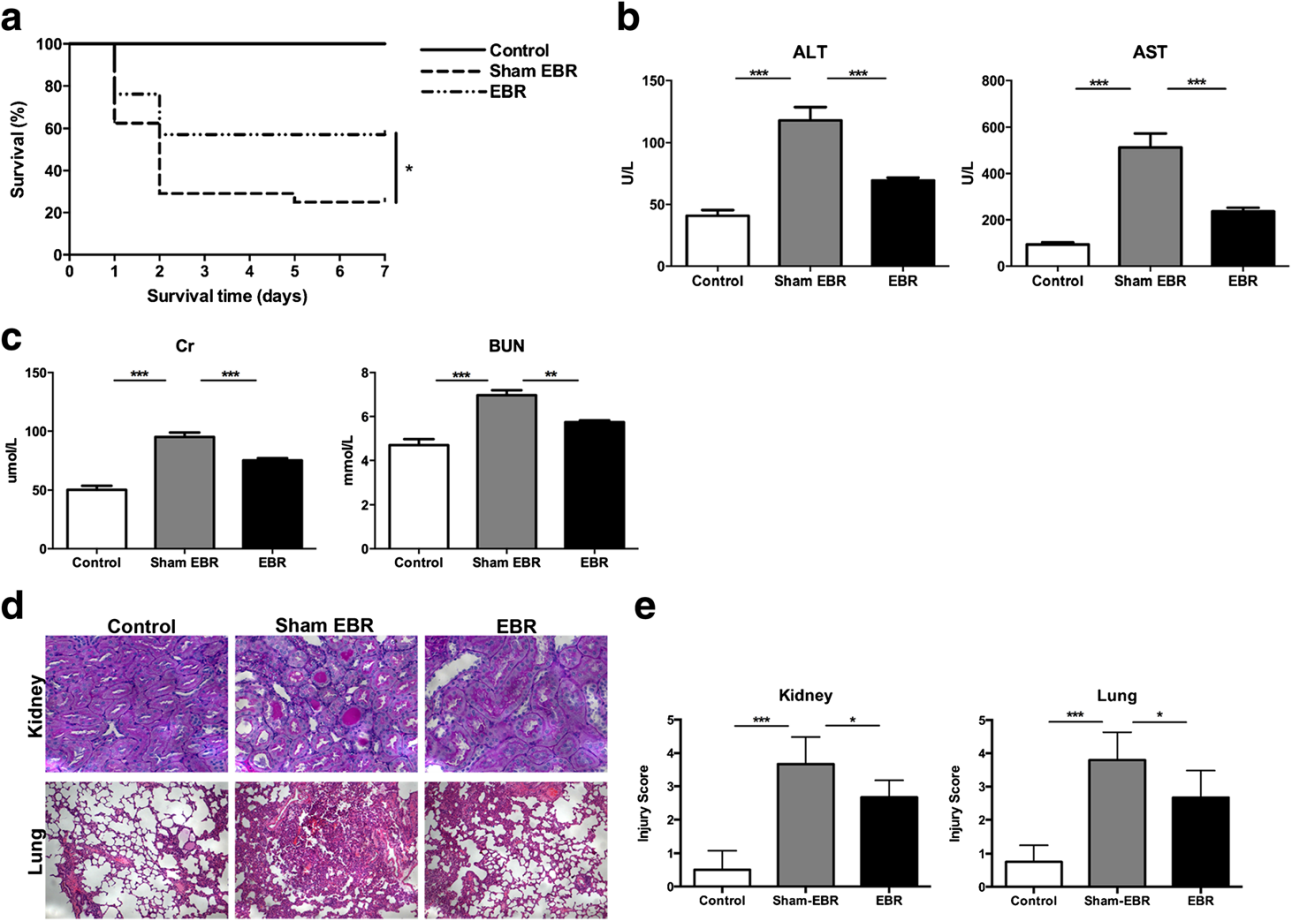
The endothelial bioreactor (EBR) system ameliorated sepsis induced by cecal ligation and puncture (CLP). **a** Survival was plotted during a 7-day period (*n* = 22–25 per group). **b** Serum ALT and AST were quantified at 48 h after CLP (*n* = 7–9 per group). **c** Serum Cr and BUN were measured at 48 h after CLP (*n* = 7–9 per group). **d** Representative photomicrographs of lung sections stained with hematoxylin and eosin (HE) and examined at ×200 magnification and of kidney sections stained with periodic acid and Schiff’s reagent (PAS) and examined at ×400 magnification. **e** Histopathologic mean of lung and kidney injury was scored at ×200 magnification at 72 h after CLP (*n* = 6 per group, at least ten fields were reviewed for each slide). *p < 0.05, **p < 0.01, ***p < 0.001. Data are expressed as mean ± SEM. *EBR* endothelial bioreactor, *CLP* cecal ligation and puncture, *ALT* alanine aminotransferase, *AST* aspartate aminotransferase, *Cr* creatinine, *BUN* blood urea nitrogen

We next investigated whether this EBR system can ameliorate sepsis. First, we analyzed survival time after CLP. We found that the EBR system can improve the survival of rats with CLP-induced sepsis. Data showed that the 7-day survival rate of sham-treated rats (sham EBR group) was only 25.0 %, while in the EBR-treated group (EBR group), it increased to 57.14 % (*p* = 0.04, sham EBR vs. EBR; Fig. 2a). Sepsis may result in multiple organ dysfunction, thus, we assessed the liver function, renal function, and histological injury of the kidney and lung. We monitored the liver function using serum ALT and AST and used serum Cr and BUN to monitor renal function. At 48 h after CLP, the levels of ALT and AST were elevated in the sham-EBR group compared with rats without induced sepsis or circulation (control group) (*p* < 0.001, sham EBR vs. control; Fig. 2b), while these metrics decreased significantly in the EBR-treated rats (*p* < 0.001, sham EBR vs. EBR; Fig. 2b). Similarly, after the EBR treatment, the levels of serum Cr and BUN also decreased (*p* < 0.01, sham EBR vs. EBR; Fig. 2c). The degree of damaged renal tubules of the kidney and the pathology of the lung that includes interstitial congestion and inflammatory cell infiltration in sham-EBR group showed much severer injuries than the control group (Fig. 2d). However, the injuries were ameliorated after the EBR treatment (Fig. 2d). Furthermore, the injury scores of the kidney (*p* < 0.05, sham EBR vs. EBR; Fig. 2e) and lung (*p* < 0.05, sham EBR vs. EBR; Fig. 2e) confirmed the protective effects of the EBR system. These data suggested that the EBR system could prolong the survival time and reduce organ injuries of CLP-induced sepsis compared with the sham EBR system.

### The EBR system alleviated inflammation in the lungs

**Fig. 3:**
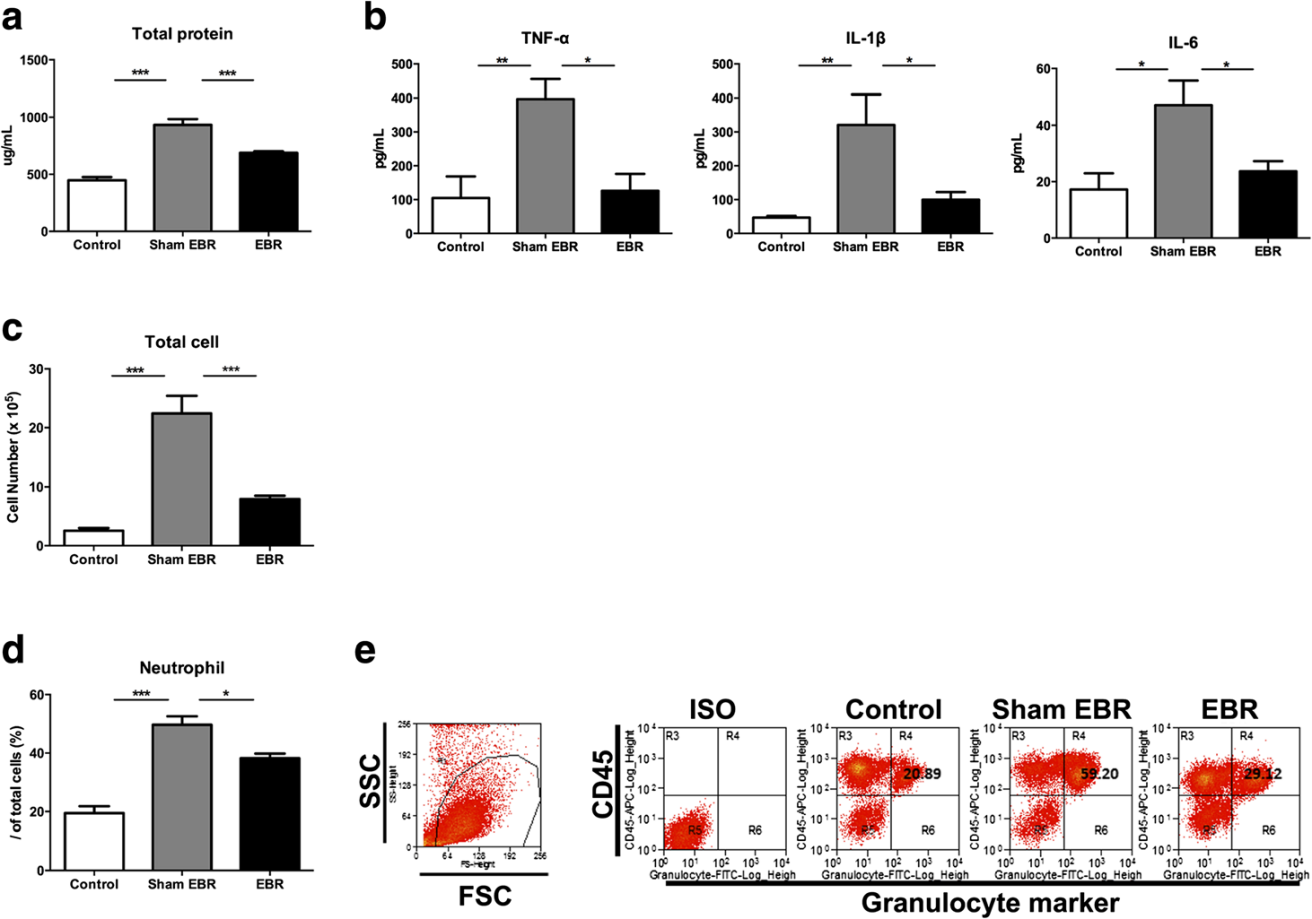
The endothelial bioreactor (EBR) system alleviated inflammation in lungs. **a** Total protein in BALF at 48 h after CLP (*n* = 7–9 per group). **b** Inflammatory cytokines in BALF were measured by ELISA at 48 h after CLP (*n* = 7–9 per group). **c** Total cell counts in BALF at 48 h after CLP (*n* = 7–9 per group). **d** The levels of neutrophils in the lung were analyzed by flow cytometer at 48 h after CLP (*n* = 7–9 per group). **e** Gating strategy for the identification of neutrophils in the lung. *p < 0.05, **p < 0.01, ***p < 0.001. Data are expressed as mean ± SEM. *EBR* endothelial bioreactor, *CLP* cecal ligation and puncture, *BALF* bronchoalveolar lavage fluid, *ELISA* enzyme-linked immunosorbent assay

The lung is one of the first organs to be affected in sepsis; cellular infiltration, along with the release of proinflammatory mediators, leads to the development of lung injury [5]. The concentration of total protein in BALF was used to assess pulmonary vascular leakage. At 48 h after CLP, the total protein levels in BALF were elevated in the sham-EBR group compared with rats without induced sepsis or circulation (control group) (*p* < 0.001, sham EBR vs. control; Fig. 3a), while the total protein decreased significantly in the EBR-treated rats (*p* < 0.001, sham EBR vs. EBR; Fig. 3a). As cytokine storm in the lungs may lead to lung injury [28], to further evaluate pulmonary inflammation in the septic rats, levels of inflammatory-associated cytokines including TNF-α, IL-1β, and IL-6 were measured in the BALF samples. We found that the levels of these cytokines increased significantly in the sham-EBR group than the control group (*p* < 0.05; Fig. 3b). EBR treatment could decrease the levels of TNF-α (*p* < 0.05), IL-1β (*p* < 0.05), and IL-6 (*p* < 0.05) compared with the sham-EBR group at 48 h after CLP (Fig. 3b). Considering that the upregulated proinflammatory cytokines can result in recruitment and activation of inflammatory cells [29], we counted the total cells in BALF of the different groups. We found that the total BALF cell count in the sham-EBR group was much more than that in the control group (*p* < 0.001; Fig. 3c). However, septic rats with EBR treatment showed a significant decrease in the total BALF cell count at 48 h after the CLP procedure compared with the sham EBR group (*p* < 0.001; Fig. 3c). In sepsis, activated neutrophils transmigrate and infiltrate into the lungs, and this overwhelming migration correlates with the severity of lung injury [30]. Thus, we examined the infiltration of neutrophils in the lungs by flow cytometer. Granulocytes marker [31] and CD45 double-positive cells were defined neutrophils in rats. We found that the infiltration of neutrophils in the lungs of EBR group was significantly reduced compared with sham-EBR group (38.2 vs. 49.7 %, *p* < 0.05; Fig. 3d, e) at 48 h after CLP. Taken together, these data suggested that the EBR system could reduce lung injury by alleviating inflammation which includes inflammation-associated cytokines, cell numbers in BALF, and the infiltration of neutrophils in the lungs.

### The EBR system modulated the function of pulmonary endothelial cells and peripheral blood neutrophils

**Fig. 4:**
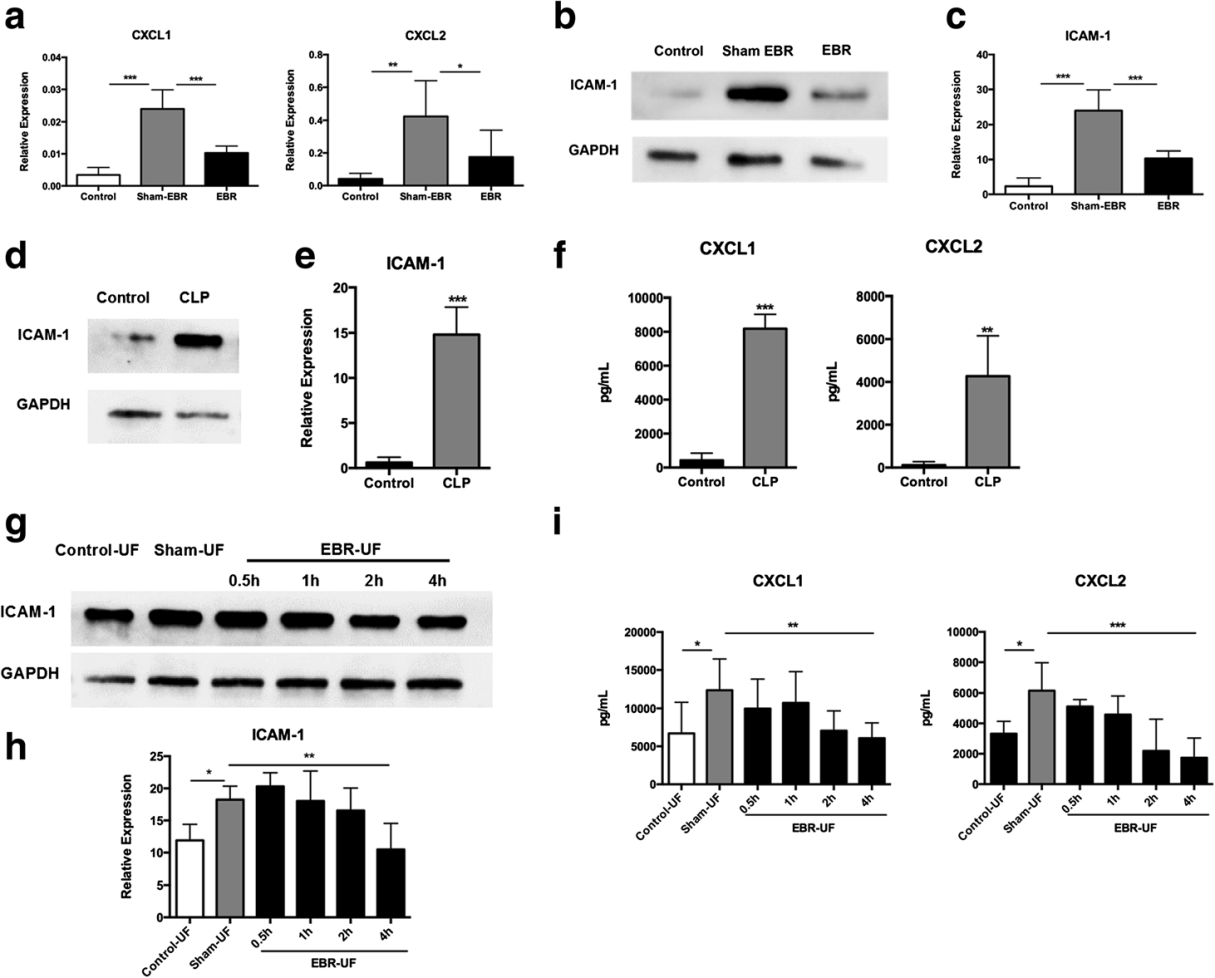
The endothelial bioreactor (EBR) system modulated pulmonary endothelial function. **a** The mRNA expression of CXCL1 and CXCL2 in the lungs was analyzed by quantitative real-time RT-PCR at 48 h after CLP (*n* = 7–9 per group). **b** The level of ICAM-1 in the lungs was examined by Western blot analysis at 48 h after CLP (*n* = 7–9 per group). **c** The mRNA expression of ICAM-1 in the lungs was analyzed by quantitative real-time RT-PCR at 48 h after CLP (*n* = 7–9 per group). **d**–**f** ICAM-1, CXCL1, and CXCL2 level of pulmonary endothelial cells from rats with or without CLP (*n* = 6 per group). **g**–**i** The EBR system was used in vitro, the UF of this system was used to stimulate pulmonary endothelial cells and the cells and supernatants were collected at 24 h after different stimuli (*n* = 6 per group). **d**, **g** The level of ICAM-1 in pulmonary endothelial cells was examined by Western blot analysis. **e**, **h** The mRNA expression of ICAM-1 in pulmonary endothelial cells was analyzed by quantitative real-time RT-PCR. **f**, **i** The levels of CXCL1 and CXCL2 in the culture supernatant of pulmonary endothelial cells were measured by ELISA. *p < 0.05, **p < 0.01, ***p < 0.001. Data are expressed as mean ± SEM. *EBR* endothelial bioreactor, *CLP* cecal ligation and puncture, *UF* ultrafiltration

Recruitment of neutrophils into the lung is a key event in the early development of acute lung injury [32]. CXCL1 and CXCL2 are dominant chemokines for attracting neutrophils in inflammatory diseases [32–34]. Thus, we analyzed the mRNA expressions of CXCL1 and CXCL2 in the lungs and found that the mRNA expressions of these two chemokines in the sham-EBR group was upregulated than the control group (CXCL1, *p* < 0.001; CXCL2, *p* < 0.01; Fig. 4a). However, the EBR system could significantly reduce the mRNA expression of CXCL1 and CXCL2 in the lung tissue compared with the sham EBR system (CXCL1, *p* < 0.001; CXCL2, *p* < 0.05; sham EBR vs. EBR; Fig. 4a). Acute inflammation is characterized by both neutrophil emigration and increased vascular permeability [35]. Activation of the vascular endothelium results in the induction of adhesion molecules (e.g., intercellular adhesion molecule-1 and E-selectin) and chemokines (e.g., CXCL1 and CXCL2) that play a central role in the cascade of leukocyte tethering, slow rolling, firm adhesion, and transendothelial migration [36, 37]. ICAM-1, a marker of pro-adhesive state endothelial cells, is highly expressed on endothelial cells in sepsis [38]. We found that the protein level of ICAM-1 (Fig. 4b) and mRNA expression of ICAM-1 (*p* < 0.001; Fig. 4c) in the sham-EBR group was upregulated than the control group, which implying the endothelial cells in the lung, were at pro-adhesive state after CLP. Furthermore, the EBR system could significantly reduce both the protein level of ICAM-1 (Fig. 4b) and the mRNA expression of ICAM-1 (*p* < 0.001, sham EBR vs. EBR; Fig. 4c) in the lung tissue compared with the sham EBR system, which suggested that the EBR system might ameliorate the pro-adhesive state. To further confirm that the EBR treatment may be involved in reducing the secretion of CXCL1 and CXCL2 and the expression of ICAM-1 of pulmonary endothelial cells, we isolated pulmonary endothelial cells from rats with or without CLP and mimicked the EBR system treatment in vitro by stimulating pulmonary endothelial cells with ultrafiltrate (UF) from control (control-UF), sham-EBR (sham-UF), and EBR group (EBR-UF). We found that both of the protein level (Fig. 4d) and the mRNA expression of ICAM-1 (*p* < 0.001; Fig. 4e) were much higher in pulmonary endothelial cells of septic rats compared with the cells isolated from healthy rats. Stimulation with UF of the sham-EBR system increased the protein level and the mRNA expression of ICAM-1 (*p* < 0.05, sham-UF vs. control-UF; Fig. 4g, h) in pulmonary endothelial cells of septic rats, whereas stimulation with UF of EBR system could reduce the level of ICAM-1 (*p* < 0.01, sham-UF vs. 4-h EBR-UF; Fig. 4g, h) in pulmonary endothelial cells of the septic rats. Meanwhile, we found that the levels of CXCL1 and CXCL2 in culture supernatant of pulmonary endothelial cells of septic rats increased significantly compared with the cells isolated from healthy rats (CXCL1, *p* < 0.001; CXCL2, *p* < 0.01; Fig. 4f). Stimulation with UF of sham-EBR system increased the levels of CXCL1 and CXCL2 (*p* < 0.05, sham-UF vs. control-UF; Fig. 4i) in culture supernatant of pulmonary endothelial cells of septic rats, whereas stimulation with UF of EBR system could reduce the levels of these two chemokines (CXCL1, *p* < 0.01; CXCL2, *p* < 0.001; sham-UF vs. 4-h EBR-UF; Fig. 4i) in pulmonary endothelial cells of the septic rats. Collectively, these data suggested that the EBR system may be involved in modulating the function of pulmonary endothelial cells and reducing the adhesion and chemotaxis of neutrophils.

**Fig. 5:**
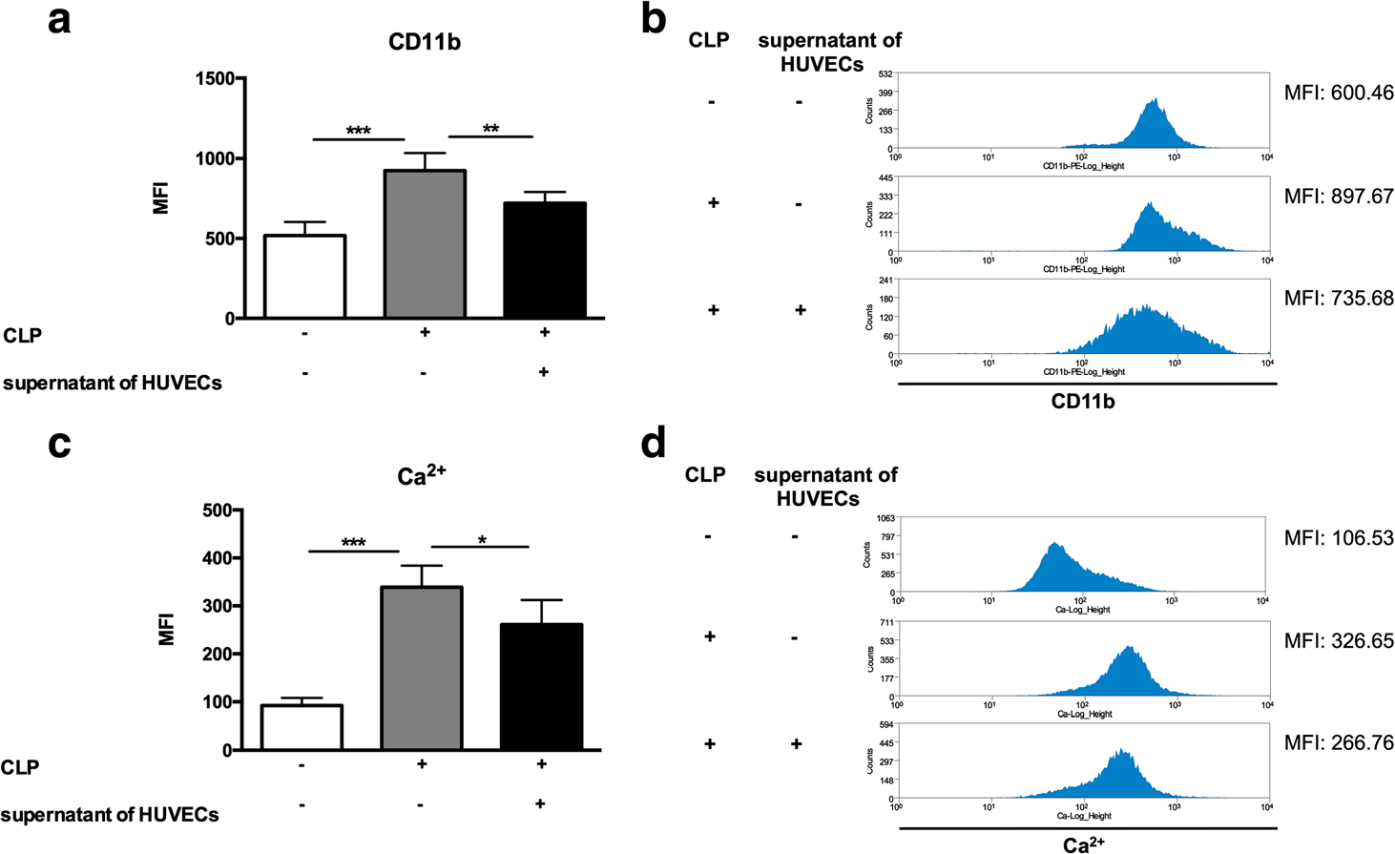
The endothelial bioreactor (EBR) system modulated the activation of peripheral blood neutrophils in vitro. **a**–**d** Peripheral blood neutrophils from rats with CLP were stimulated with culture supernatant of HUVECs for 4 h, and the supernatant were collected at 24 h after this stimulation. **a**, **b** The mean fluorescence intensity of CD11b was analyzed by flow cytometer (*n* = 7–9 per group). **c**, **d** The mean fluorescence intensity of intracellular free calcium was analyzed by flow cytometer (*n* = 7–9 per group). *p < 0.05, **p < 0.01, ***p < 0.001. Data are expressed as mean ± SEM. *EBR* endothelial bioreactor, *CLP* cecal ligation and puncture, *MFI* mean fluorescence intensity, *HUVECs* human umbilical vein endothelial cells

In addition, excessive activation of neutrophils is a major component of tissue damage and organ failure in sepsis [39]. CD11b and intracellular free calcium level are parameters commonly used for the assessment of neutrophil activation [40, 41]. Therefore, we isolated peripheral blood neutrophils from rats with CLP and stimulated with culture supernatant of HUVECs for 4 h. The control groups were neutrophils without any stimulation. Then, the levels of CD11b and intracellular free calcium were analyzed by flow cytometer. The mean fluorescence intensity (MFI) of CD11b and the level of intracellular free calcium of peripheral blood neutrophils from CLP-rats increased significantly compared with neutrophils from healthy rats (*p* < 0.001, CLP+ and supernatant of HUVECs− vs. CLP- and supernatant of HUVECs−; Fig. 5a–d). However, these two parameters of neutrophils from septic rats decreased after stimulated with culture supernatant of HUVECs for 4 h compared with neutrophils with no stimulation (CD11b, *p* < 0.01; Ca^2+^, *p* < 0.05; CLP+ and supernatant of HUVECs+ vs. CLP+ and supernatant of HUVECs−; Fig. 5a–d). These data show that the EBR system may be involved in modulating the activation of peripheral blood neutrophils.

## Discussion

A previous study by our laboratory has showed that timely EBR therapy improves cardiovascular performance and prolongs the survival time of endotoxemia sepsis in swine [15]. In the present study, we established a new EBR system for septic rats and demonstrated that the EBR system ameliorated CLP-induced sepsis and improved survival and organ functions. In animal models, the cytokine response is fully activated by 16–18 h after CLP [42], and the ligated cecum begins to heal after 24 h [19, 43]. In clinical practice, patients often do not come to the hospital at the early stage of infection. In order to simulate clinical practice, we started the treatment at 18 h after CLP. The therapeutic use of extracorporeal bioreactors has a long tradition. Different cell-based extracorporeal organ support systems involving either hepatocytes or renal tubular cells have been used against acute liver failure [44–46] and acute renal failure associated with sepsis [9, 47]. An extracorporeal bioartificial kidney consisting of a conventional hemofilter connected to a renal tubule assist device has shown its ability to successfully replace the filtration, transport, metabolic, and endocrine functions of the kidney in critically ill patients with acute renal failure in phase I/II clinical trials [10, 47]. Moreover, an extracorporeal treatment with donor granulocytes appears to be well tolerated and shows promising efficacy results in patients with septic shock, thus encouraging further studies [48].

The EBR system is an extracorporeal cell therapy with endothelial cells. We found that the EBR system alleviates inflammation in the lungs and reduces the infiltration of inflammatory cells, especially neutrophils. In sepsis, a number of different mechanisms may induce functional modulation of the endothelium, including increased expression of cell adhesion molecules and trafficking of leukocytes [49]. Endothelial cells are critical in maintaining a delicate balance between vasoconstriction and vasodilation, blood cell adherence and non-adherence, anticoagulation and procoagulation, and permeability and tightness [50]. These cells are not inert, rather they may adapt their function upon interaction with inflammatory mediators, and these changes are referred to as activation. They encompass a change from an anti- into a procoagulant surface; the expression of adhesion molecules; the production of inflammatory mediators, including chemoattractant agents; and the production of vasoactive compounds [11]. Two studies in cell-based treatments showed that transplanting endothelial cells to mice with ischemic acute kidney injury could change the function of renal endothelial cells and improve the renal function [51, 52]. The dysfunction or activation of endothelial cells plays a major role in the pathogenesis of sepsis [11, 14, 50]. Thus, endothelial cells represent an attractive therapeutic target in sepsis.

ICAM-1 is a cell-surface protein that is expressed at very low levels on pulmonary endothelium. The expression of ICAM-1 is upregulated after stimulation by inflammatory mediators such as cytokines and bacterial lipopolysaccharides during septic processes [53, 54]. ICAM-1 mediates inflammatory responses via adhesion of leukocytes to activated endothelium and subsequent leukocyte transmigration through the pulmonary endothelial layer [55]. The increased expression of endothelial adhesion molecules either at the membrane level or in the plasma typifies different models of sepsis [12]. Moreover, there is a close relationship between plasma levels of adhesion molecules and outcomes of sepsis. In human sepsis, studies have shown that the higher the plasma levels of ICAM-1, the greater the number of organs damaged and the mortality [13, 56]. The present study showed that the level of ICAM-1 in the lung tissue was increased after CLP, while the EBR treatment reduced its expression compared with the sham treatment. Furthermore, the level of pulmonary endothelial ICAM-1 significantly decreased after the EBR treatment in vitro. In other words, our findings indicate that this extracorporeal endothelial therapy may be involved in reducing the expression of ICAM-1 in the pulmonary endothelium, and this change should help to alleviate the adhesion of neutrophils. Moreover, in vivo and in vitro, we also found that this treatment decreased the secretion of CXCL1 and CXCL2 of septic pulmonary endothelial cells compared with the sham treatment. CXCL1 and CXCL2 are important neutrophil-attracting chemokines. Recent studies showed that CXCR2 (the receptor of CXCL1 and CXCL2) blockade results in impaired neutrophil recruitment in lipopolysaccharide-induced acute lung injury [57, 32]. Our data indicate that this EBR therapy may be involved in reducing the chemotaxis of neutrophils in the lung tissue via decreasing the secretion of CXCL1 and CXCL2 of pulmonary endothelial cells. Neutrophils are known to play an important role in inflammatory responses [58]. However, excessive activation of neutrophils is a direct cause of tissue damage and organ failure in sepsis [39, 58]. Mac-1 (CD11b/CD18), the major subtype of integrins, is responsible for the firm adhesion of neutrophils to endothelium [40]. Once the neutrophils are activated, the shape of these cells changed and the amount of Mac-1 increased, resulting in enhanced adhesion to the endothelium and transmigration and infiltration of these neutrophils [40]. Another parameter commonly used for the assessment of neutrophil activation is intracellular free calcium level. Ca^2+^-dependent functions include activation of the membrane-associated superoxide-generating electron transporter, NADPH oxidase, and adhesion to the endothelium [41]. The present study showed that the EBR system decreased the CD11b expression and intracellular free calcium level of peripheral blood neutrophils compared with the sham treatment, which might modulate the activation of these neutrophils.

In the early phase of sepsis, the overwhelming inflammatory response is initiated after microbial infection [59], while the late phase is characterized by T cell hyporesponsiveness and defective antigen presentation [60, 61]. This phase is considered a state of immunosuppression or immunoparalysis of the host [62]. Massive apoptosis of lymphocytes is one of the main drivers leading to immunoparalysis [63]. To assess the adaptive immune response, circulating and splenic levels of CD4^+^ helper T cells, CD8^+^ cytotoxic T cells, and CD4^+^CD25^+^Foxp3^+^ T regulatory cells were measured at 72 h after CLP. However, there was no significant difference between EBR-treated and sham-treated rats either in the peripheral circulation or in the spleen (Additional file 1: Figure S1). Thus, the 4-h EBR system treatment had no effect on the levels of T lymphocytes in the current rat model.

## Conclusions

In summary, these data suggested that EBR system ameliorates CLP-induced sepsis and improves survival and organ functions compared with the sham EBR system. The EBR system alleviates inflammation in the lungs and reduces the infiltration of inflammatory cells and may be involved in modulating the function of pulmonary endothelial cells and reducing the adhesion and chemotaxis of neutrophils. In the same time, the EBR system might probably modulate the activation of peripheral blood neutrophils directly.

## Additional file

Additional file 1: Figure S1. The endothelial bioreactor (EBR) had no effect on the levels of T lymphocytes in septic rats. Circulating and splenic levels of CD4^+^ helper T cells, CD8^+^ cytotoxic T cells, and CD4^+^ CD25^+^ Foxp3^+^ T regulatory cells were analyzed by flow cytometer at 72 h after CLP (*n* = 7–9 per group). EBR = endothelial bioreactor; CLP = cecal ligation and puncture. (TIF 307 kb)
