# Supplementary figures and images for: Endothelial bioreactor system ameliorates multiple organ dysfunction in septic rats
Source: Intensive Care Med Exp. 2016 Jul 22;4:23. doi: 10.1186/s40635-016-0097-y (PMC4958089; doi:10.1186/s40635-016-0097-y)

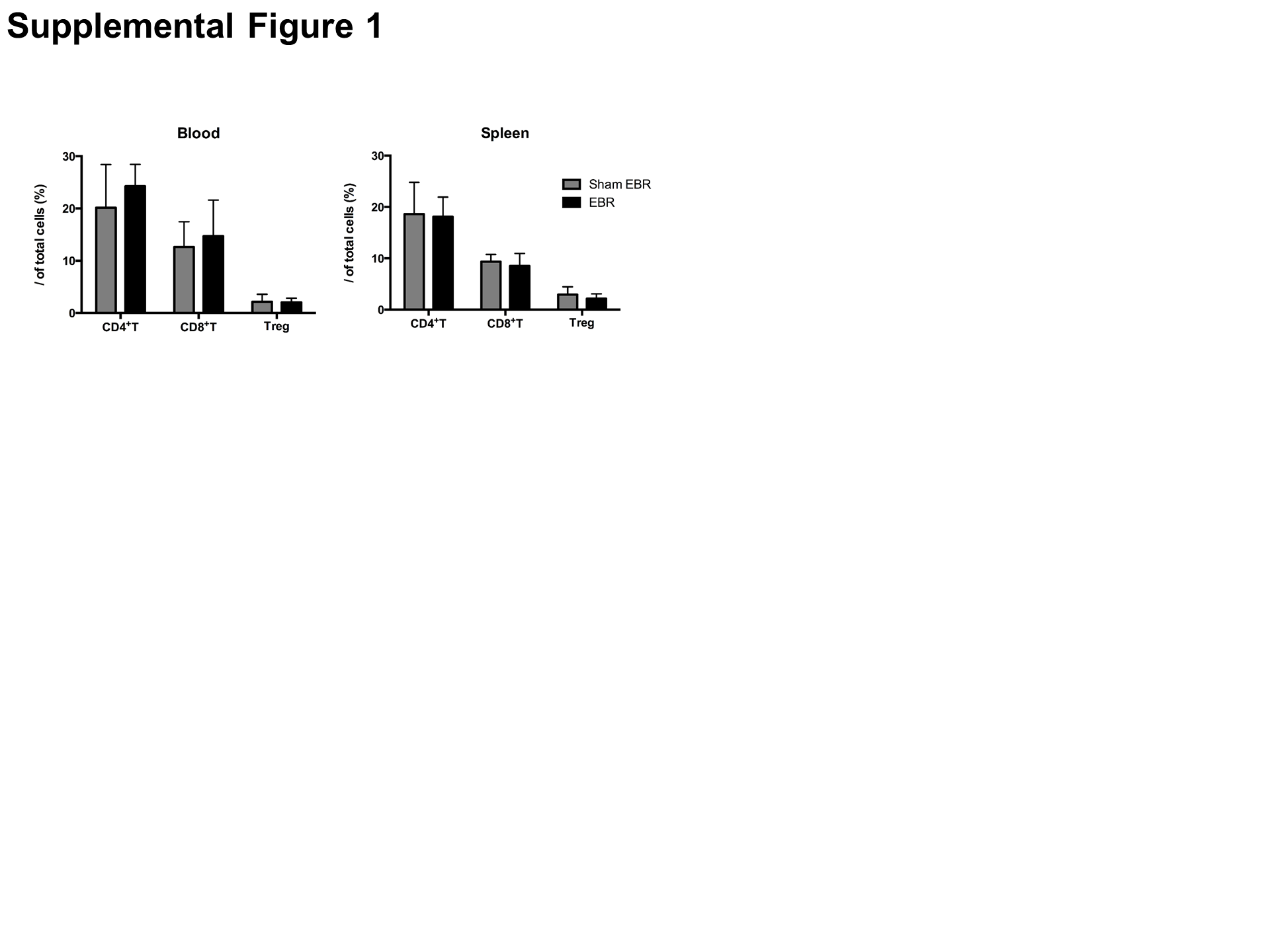

Supplement: Additional file 1: Figure S1. — The endothelial bioreactor (EBR) had no effect on the levels of T lymphocytes in septic rats. Circulating and splenic levels of CD4+ helper T cells, CD8+ cytotoxic T cells, and CD4+ CD25+ Foxp3+ T regulatory cells were analyzed by flow cytometer at 72 h after CLP (n = 7–9 per group). EBR = endothelial bioreactor; CLP = cecal ligation and puncture. (TIF 307 kb) [file 40635_2016_97_MOESM1_ESM.tif]
